# Loss of SIM2s inhibits RAD51 binding and leads to unresolved replication stress

**DOI:** 10.1186/s13058-019-1207-z

**Published:** 2019-11-27

**Authors:** Scott J. Pearson, Jessica Elswood, Rola Barhoumi, Brittini Ming-Whitfield, Monique Rijnkels, Weston W. Porter

**Affiliations:** 1 Department of Integrative Biosciences, College of Veterinary Medicine, Texas A&M University, College Station, TX 77843 USA; 2 Department of Molecular and Cellular Medicine, Texas A&M University, College Station, TX 77843 USA; 3 Present Address: Veterinary Integrative Biosciences, Texas A&M University, College of Veterinary Medicine, College Station, TX 77843 USA

**Keywords:** Breast cancer, Tumor suppressor, Genetic instability, SIM2, Replication stress

## Abstract

**Background:**

Mutations in genes associated with homologous recombination (HR) increase an individual’s risk of developing triple-negative breast cancer (TNBC). Although known for their role in repairing dsDNA breaks, HR repair elements also stabilize and restart stalled replication forks. Essential to these functions are RAD51 and its paralogs, each of which has a unique role in preventing replication fork collapse and restart. However, progress toward understanding the regulation of these factors has been slow. With such a pivotal role in the maintenance of genomic integrity, furthering our understanding of this pathway through the discovery of new factors involved in HR is important. Recently, we showed that singleminded-2s (SIM2s) is stabilized in response to dsDNA breaks and is required for effective HR.

**Methods:**

Initial analysis of the effect loss of SIM2s has on replication stress resolution was conducted using DNA combing assays in established breast cancer cell lines. Further analysis was conducted via immunostaining to determine the effect loss of SIM2s has on factor recruitment. In vivo confirmation was achieved through the use of a mammary epithelial cell conditional knockout mouse model before SIM2s’ role in RAD51 recruitment was determined by immunoblotting.

**Results:**

Here, we show loss of SIM2s decreases replication fork stability, leading to fork collapse in response to genotoxic stress. Furthermore, loss of SIM2s results in aberrant separation of sister chromatids during mitosis, which has been previously shown to result in chromosomal fragmentation and aneuploidy. Interestingly, loss of SIM2s was shown to result in failure of RAD51 to localize to sites of replication stress in both breast cancer cell lines and primary mammary epithelial cells. Finally, we observed SIM2 is stabilized in response to genotoxic stress and interacts with RAD51, which is necessary for RAD51-DNA binding.

**Conclusions:**

Together, these results show a role for SIM2s in the resolution of replication stress and further characterize the necessity of SIM2s for effective RAD51 loading in response to DNA damage or stress, ultimately promoting genomic integrity and thus preventing the accumulation of cancer-promoting mutations.

## Background

Mutations in components of the homologous recombination (HR) pathway have long been associated with an increased risk of developing breast cancer. More specifically, mutations in the DNA-damage repair (DDR) gene BRCA1 alone can increase the probability of developing breast cancer before the age of 80 from 12 to 75% [1, 2]. Moreover, individuals with BRCA1/2 mutations are significantly more likely to develop highly invasive/malignant triple-negative breast cancer (TNBC). In fact, 42% of breast cancer cases in BRCA1 mutation carriers are TNBC compared to 15–20% in non-BRCA-mutated breast cancers [3, 4]. Although this increased risk for TNBC could be attributed to deficiencies in DDR, novel roles for BRCA1 also include the stabilization and resolution of stalled replication forks arising from a multitude of different factors [5]. With the increased instance of highly invasive breast cancer in individuals with mutations in BRCA, the identification of other factors that mimic the ability of BRCA1 to maintain genomic stability would expand our repertoire of oncogenic markers and increase our ability to design targeted treatments for breast cancer patients. This would help to define malignancies that are more likely to become invasive and may respond to PARP inhibitor (PARPi) and platinum salt therapeutics, which are becoming the standard of care for individuals with BRCA mutations.

Replication stress can be induced by many different aberrations during DNA replication; however, it can generally be defined as a slowing or stalling of the replication fork complex [6]. Endogenously, replication stress can be caused by unrepaired DNA lesions, ssDNA, unusual DNA structures (such as hairpins and triplexes), transcription, mis-incorporation of nucleotides, and limited resources, to name a few [6]. With many potential causes of replication stress, there is no singular replication stress repair pathway. Interestingly, a common factor in replication stress stabilization and repair after prolonged stalling of replication forks is the accumulation of non-DNA-damage-associated RAD51 and other members of the HR pathway. This suggests a recombination-based attempt to resolve the stalled fork [7–11].

During the S phase, BRCA1 has been shown to protect stalled replication forks from collapse, preventing dsDNA breaks that can lead to the development of detrimental mutations [10]. Later in the cell cycle during the onset of mitosis, sister chromatids are intertwined and are separated via a topoisomerase II-dependent process [12]. Failure of this process to occur can lead to chromosomal breakages, potentially resulting in aneuploidy or cell death. RAD51 plays a role in this replication process during replication restart after stalling [7]. Early in hydroxyurea (HU)-induced replication stress, low levels of RAD51 are associated with nascent ssDNA at the replication fork in a XRCC3-dependent manner [7]. RAD51 is predicted to play a role in the quick restart of stalled replication forks, as depletion of RAD51 leads to the persistence of stalled forks even after HU has been removed [7]. However, in cells with forks stalled for longer than 24 h, fork restart does not occur after removal of HU, and instead, RAD51 foci formation occurs. This suggests that after prolonged stress, RAD51 plays a role in the removal and repair of stalled and collapsed forks [7].

Recently, our lab demonstrated a role during HR for singleminded 2s (SIM2s; a short splice variant of SIM2, and the predominant isoform of SIM2 in the mouse mammary gland) [13, 14]. SIM2s is a member of the basic-helix-loop-helix/PER-ARNT-SIM family of transcription factors. In its role in HR, SIM2 is phosphorylated and stabilized in response to ionizing radiation, which can be abrogated through the mutation of a serine residue located within an ATM (ataxia telangiectasia mutated) consensus site [13]. Loss of *SIM2* results in reduced recruitment of RAD51 to sites of DNA damage and, thus, an overall decrease in HR efficiency [13]. In addition to playing a role in HR, loss of *SIM2s* has been associated with an epithelial mesenchymal transition (EMT) in both normal breast and malignant cell lines [14–20]. Moreover, loss of *SIM2* or the introduction of a point mutation at S115, a likely target of ATM-dependent phosphorylation, in a xenograft model results in a significant increase in metastasis found within the lung [13, 17]. Here, we propose a role for SIM2s in maintaining genomic stability by assisting the resolution of prolonged replicative stress.

## Methods

### Cell culture

SUM159 and MCF7 cells were obtained from American Type Culture Collection (ATCC) and maintained according to the ATCC guidelines.

### Generation of cell lines

Cell lines were generated as previously described [13]. In brief, SIM2 constructs were generated via long cDNA synthesis. Plasmids were amplified using Subcloning Efficiency™ DH5α™ competent cells (Life Technologies). Plasmid DNA was isolated using the HiPure Plasmid Maxiprep kit (Life Technologies) or the ZymoPURE Plasmid DNA Isolation Kit (Zymo Research). Ten micrograms of plasmid was mixed with GeneJuice (EMD Millipore) in 1 mL of Opti-MEM (Life Technologies) and incubated at room temperature for 15 min. This mixture was then added onto Phoenix-AMPHO lentiviral packaging cells (ATCC). Cells were incubated for 24 h at 32 °C and 5% CO_2_. Media was collected and filtered through a 0.45-μm filter. The recommended amount of Sequabrene (Sigma) was added to the filtered media. The media was then added to SUM159 cells in six-well plates. Plates were centrifuged at 200×*g* for 60 min and allowed to incubate overnight at 32 °C and 5% CO_2_. Media was again collected from the packaging cells the next day, and target cells were transduced a second time, as described above. Puromycin selection (2 μg/mL) was started the following day and maintained for at least a week [14].

### Generation of shSIM2 containing cell lines

MCF7 cells containing *shSIM2* were previously established [14]. In brief, the *shSIM2* was generated by inserting 5′ - GAT CCG GTC GTT CTT TCT TCG AAT TTC AAG AGA ATT CGA AGA AAG AAC GAC CTC TTT TTT GGA AA-3′ into pSilencer U6-retro 5.1 shRNA vector (Ambion), and control cells (*pSIL*) were generated by inserting a nonspecific scrambled sequence into the same vector. Plasmids were then packaged into lentivirus using Phenix HEK293-Ampho packaging cells as previously described [14].

### Primary mammary epithelial cell (MEC) isolation

Primary MECs were isolated from the #3, #4, and #5 mammary gland tissues and placed in wash buffer (1× DMEM/F12 (Life Tech), 5% FBS (Atlanta Biological), 50 μg/mL (Life Technologies)) and mechanically homogenized with #10 scalpels (Feather). Glands were then placed in 2 mg/mL Collegenase A (Roche) in wash buffer and incubated at 37 °C with shaking for ~ 1.5 h. Organoids were pelleted at 600×*g* for 10 min, and supernatant was aspirated. Free nucleic acids were then digested with DNAseI treatment (100 μg/mL DNAse (Sigma), DMEM/F12). Organoids were washed in wash buffer four times and subsequently pelleted by pulse spinning at 450×*g*. Organoids were then digested in 1 mg/mL trypsin (Life Technologies) at 37 °C for ~ 20 min before being brought up to 10 mL in growth media (DMEM/F12, 10% FBS, 100 units/mL penicillin/streptomycin (Life Technologies), 5 μg/mL insulin (Sigma), 50 μg/mL gentamicin (Life Technologies), 1 μg/mL hydrocortisone, 10 ng/mL mouse epidermal growth factor (EGF; Life Technologies)), and single cells were pelleted at 450×*g* for 3 min. MECs were washed twice more in growth media and pelleted again. MECs were finally plated on 10-cm tissue culture dishes and cultured at 32 °C and 5% CO_2_.

### Antibodies

Antibodies and concentrations are listed in Additional file 1: Table S1.

### DNA combing assay

DNA combing assays were performed as previously described using IdU (Sigma) and CldU (Sigma) with the indicated modifications in timepoints [21]. In brief, the cells were dosed with the indicated reagents (IdU, CldU, HU, DMSO), at the indicated dosages, for the indicated amounts of time depending on the experiment being conducted. Cells were then washed with PBS and trypsinized and collected in a 15-mL conical tube before being washed again with ice-cold PBS, brought to a concentration of 400 cells/μL and placed on ice. Two microliters of cells was then pipetted onto a charged microscope slide and allowed to dry almost completely. Fifteen microliters of lysis solution (0.2 M Tris pH 7.4, 50 mM EDTA, 0.5% SDS) was added, and slides were incubated at room temperature for 10 min. Slides were then tilted to a 25° angle, allowing DNA fibers to run down slide, and allowed to dry completely. DNA was then fixed in a 3:1 methanol to acidic acid solution for 2 min, and then removed and allowed to dry overnight.

The next day, slides were placed at − 20 °C and incubated for a minimum of 24 h before proceeding to the next step. Slides were then treated with 2.5 M HCl for 30 min, washed with 0.1% PBST (PBS-Tween) for 3 min, and then incubated in PBS two times for 3 min. Slides were blocked in 5% BSA (bovine serum albumin) for 30 min. DNA was then probed with the indicated primary antibodies for 1 h, before washing two times with PBS for 3 min each. Finally, a secondary antibody was added and incubated for 1 h. Slides were washed two more times in PBS for 3 min and then images were captured using a Zeiss 780 confocal microscope, and fiber lengths were measured in ImageJ.

### Anaphase bridges

Cells were maintained at 37 °C and 5% CO_2_. First, cells were synchronized using a di-thymidine block. Briefly, cells were incubated in 2 mM thymidine (Cayman Chemical) for 19 h, washed, and cultured again in normal media for 9 h. Afterwards, 2 mM thymidine was reapplied for an additional 17 h. Cells were washed again, and normal media was added for 9 additional hours. Finally, cells were fixed with 4% paraformaldehyde (Santa Cruz) and stained with Hoescht 33342 (Life Technologies). Images were captured using a Zeiss 780 confocal microscope.

### Immunofluorescent (IF) staining of cells

IF was conducted as previously described [14]. Images were captured using a Zeiss 780 confocal microscope. Quantification of nuclear intensity was done in ImageJ.

### Immunostaining of tissue sections

IF of tissue sections was performed as previously described [20]. Images for analysis were captured on a Zeiss Axio Imager.Z1, and representative images were captured on a Zeiss 780 confocal microscope. Quantification of nuclear intensity was done in ImageJ.

### Immunoblotting

Immunoblotting was done as previously described [13].

### Cell fractionation

Cell fractionation was performed as previously described [22] with the following modification: chromatin was fragmented using a bioruptor pico (Diagenode) with 30× 1-min sonication intervals.

### Co-immunoprecipitation

All steps were conducted on ice or at 4 °C. All beads were washed three times with five volumes TBS before use. Cells were lysed in RIPA buffer containing 1 mM Na_3_VO_4_ (Sigma) and 1 mM complete ULTRA tablets mini EDTA-free Easy pack (Roche) and agitated for 30 min prior to centrifugation at 10,000×*g* for 10 min. Protein concentrations were determined via DC protein assay (Bio-Rad), and 100 μg of protein was added to IgG control beads (Cell Signaling, 5873S or 8726S) or 6 μg of the indicated antibody before incubating overnight. Magnetic beads (Active Motif, 53,033) were then added to the antibody/protein mixture and allowed to incubate for an additional 4 h. Tubes were then placed on a magnetic separator, and beads were washed three times with TBS before being resuspended and boiled for 5 min in 2× Laemmli sample buffer lacking reducing agent. β-mercaptoethanol was then added, and samples were again boiled for 5 min before immunoblotting.

### RNA isolation and real-time qPCR (RT-qPCR)

RNA isolation, reverse transcription, and RT-qPCR were performed as previously described [17]. Gene expression was evaluated with the following primers: *Sim2s*, 5′-AACCAGCTCCCATGTTTGAC-3′ (forward), 5′-ACTCTGAGGAACGGCGAAAA-3′ (reverse) and *Actb*: 5′-GCAACGAGCGGTTCC G-3′ (forward), 5′-CCCAAGAAGGAAGGCTGGA-3′ (reverse). Expression was determined using the 2^−ΔΔCt^ method and normalized relative to *Actb*.

### Statistical analysis

All experiments were done in biological triplicates with technical duplicates at a minimum and repeated three times while scientists were blinded to group identity. Before conducting two-tailed Student’s *t* tests, normal distribution was confirmed, and likelihood ratio and Pearson’s statistical test were used for goodness of fit comparisons. Significance was considered at *p* < 0.05.

### Study approval

Animal studies were approved by the Texas A&M University Laboratory Animal Care Committee in accordance with IACUC guidelines.

## Results

### Loss of SIM2s leads to an increase in replication fork collapse but does not affect replication restart speed

**Fig. 1:**
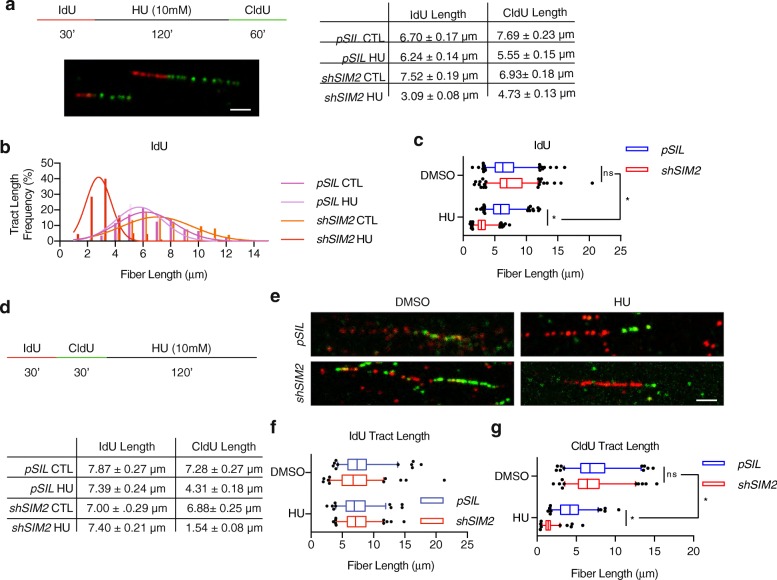
Loss of SIM2s leads to an increase in replication fork collapse. **a** Illustration of experimental design and raw measurements collected from DNA combing assays in MCF7-*shSIM2* and MCF7-*pSIL* for experiments in **b** and **c**. **b**, **c** Visualization of IdU tract lengths from MCF7-*shSIM2* and MCF7-*pSIL* cells treated with 10 mM HU or no treatment. **d** Illustration of experimental design and raw measurements for DNA combing assays for **f** and **g**. **e** Representative images enhanced for clarity of DNA tracts isolated from the indicated treatment group. **f** Visualization of IdU tract lengths from MCF7-*shSIM2* and MCF7-*pSIL* cells treated with DMSO or 10 mM HU. **g** Visualization of CldU tract lengths from MCF7-*shSIM2* and MCF7-*pSIL* cells treated with DMSO or 10 mM HU. Values indicate the median with the interquartile range. Whiskers span 5-95th percentile; *n* ≥ 100 strands. Student’s *t* test was performed to test significance. All scale bars, 1 μm. *p value < 0.05

It has been previously demonstrated that members of the HR DDR pathway are associated with maintaining genomic stability through the resolution of replicative stress [5, 7–11]. Having recently discovered SIM2s as a novel protein involved in HR, we hypothesized that loss of *SIM2s* (through inclusion of *shSIM2)* would result in a decrease in genomic stability [13]. To test this, we pulse labeled our previously established MCF7-*shSIM2* and MCF7-*pSIL*-*scrambled* cell lines with the thymidine analog IdU (5-Iodo-2′-deoxyuridine) for 30 min. Cells were then washed, and the control groups were immediately treated with CldU (5-chloro-2′-deoxyuridine) for the indicated time in order to establish a baseline tract length for unperturbed cells. At the same time, treatment groups were treated with HU (a potent antineoplastic agent that inhibits DNA replication through the inhibition of ribonucleoside diphosphate reductase [RNR]) for 2 h. Finally, treatment groups were pulse labeled for the indicated time with CldU (Fig. 1a) [13, 14]. A minimum of 100 tracts were measured for each group and analyzed for overall length (Fig. 1a). To correct for any differences in replication speed between cells containing *shSIM2* and those containing *pSIL-scrambled*, tract lengths of HU-treated groups were normalized to their untreated, basal counterparts for statistical analysis. Using this method, we were able to assess changes in both replication restart speed and replication fork stability by measuring the IdU and CldU tract lengths, respectively.

We first analyzed the replication restart speed of *shSIM2*-containing cells by measuring the tract length of CldU. Here, longer tract lengths correlate with a shorter amount of time for replication restart to occur after release from replication stress, as this would allow the cell more time to synthesize nascent DNA and incorporate CldU. Interestingly, when we assessed DNA replication restart 30 min after HU release, no visible CldU tracts were observable (data not shown), leading us to extend CldU pulse labeling to 60 min. However, after extending the CldU pulse time, we found no significant change in replication restart between *shSIM2* and control groups (Additional file 1: Figure S1 A, B).

Next, we analyzed replication fork stability by measuring the IdU tract length. In the case that replication forks become unstable during HU treatment, they will collapse, leading to a shortening of the IdU tract. This shortening of the tract length of replicated DNA has been previously attributed to failure of a cell to maintain the stability of the stalled replication fork, leading to its collapse and subsequent re-replication [7]. In this way, we were able to observe a significant decrease in replication fork stability in cells containing *shSIM2* by measuring a significant decrease in IdU tract length in these cells after treatment with HU (Fig. 1b, c).

To confirm this finding, we again pulse labeled MCF7-*shSIM2* and MCF7-*pSIL* control cells with IdU for 30 min, and then immediately pulse labeled with CldU for another 30 min before treating cells for 2 h with DMSO or 10 mM HU (Fig. 1d, e). We then measured the length of IdU tracts and CldU tracts that were immediately adjacent to IdU tracts, which eliminated any newly firing forks (Fig. 1f). A slight decrease in IdU replication length was observed in MCF7-*shSIM2* cells treated with DMSO, suggesting there may be differences in replication speeds between the two cell lines, which we again corrected for by normalizing the tract length of HU-treated groups to their untreated, basal counterparts for statistical analysis (Fig. 1f). Of note, we found a significant decrease in the CldU tract length in MCF7-*shSIM2* cells treated with HU, supporting our findings that loss of *SIM2* results in a significant increase in replication fork collapse (Fig. 1g).

### Loss of SIM2s leads to an increase in stalled forks and newly firing origins

**Fig. 2:**
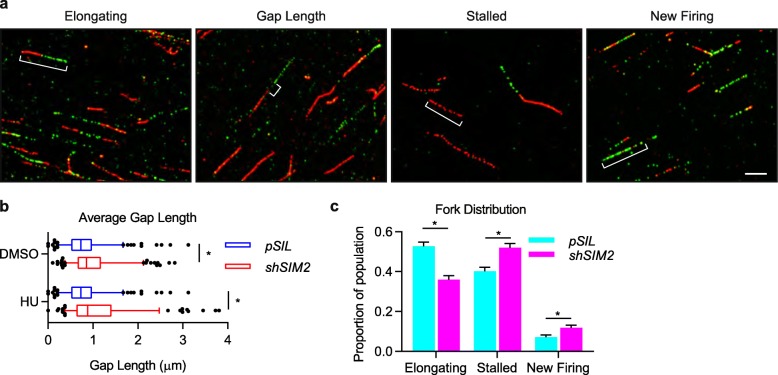
Loss of SIM2s increases the incidence of aberrations associated with stalled replication forks. **a** Representative images of DNA combing assays demonstrating the indicated conditions. Scale bar, 2 μm. **b** DNA fiber tracts isolated from MCF7 cells that were treated as in Fig. 1a were assessed for the presence of gaps between IdU and CldU tracts. A significant increase in gap length was observed in cells containing *shSIM2*, with no difference found between no treatment and HU treatment groups. Values indicate the median with the interquartile range. Whiskers span 5-95th percentile; *n* ≥ 100 strands. Student’s *t* test was performed to test significance. **c** Finally, *shSIM2* containing cells had lower incidence of actively elongating tracts, with a significant increase in stalled forks and newly firing origins. Likelihood ratio and Pearson’s chi-squared tests were performed to test correlations; *n* ≥ 100 strands. *p value < 0.05

It has been reported that stalled or collapsed replication forks can lead to formation of a gap between IdU and CldU labeling, possibly due to the firing of a new origin downstream of the stalled fork [23]. Upon measuring the length of gaps between the two pulse labels (from Fig. 1a), we found that, although there is no difference between treated and untreated cells, cells lacking SIM2s had significantly larger gaps than control cells (Fig. 2a, b). As larger gap sizes have been attributed to multiple causes, we next tested MCF7-*shSIM2* and control cells for the frequency of elongating replication forks, stalled replication forks, and newly firing origins (Fig. 2a, c) [23]. Cells containing *shSIM2* exhibited a higher frequency of both stalled replication forks, as well as an increase in the presence of newly firing replication forks. Both of these findings suggest that loss of SIM2s leads to genomic instability that culminates in the inability to resolve replication stress.

### Loss of SIM2s disrupts DNA replication

**Fig. 3:**
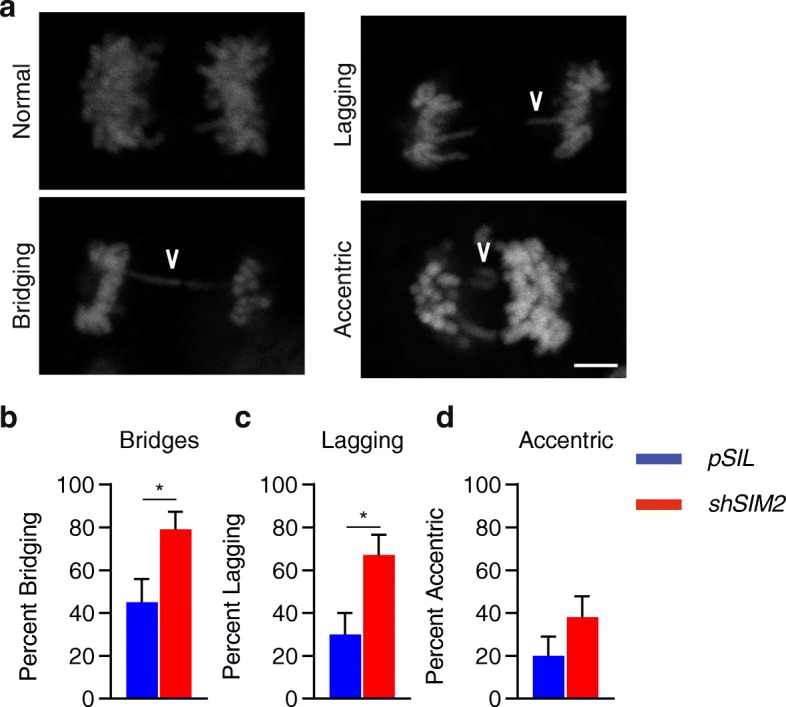
Loss of SIM2s disrupts DNA replication. **a** Representative images showing normal, bridging, lagging, and acentric chromosomes during mitosis. MCF7-*shSIM2* and MCF7-*pSIL* cells were synchronized and fixed during anaphase before being analyzed for the presence of **b** bridging strands, **c** lagging strands, and **d** acentric chromosomes. Likelihood ratio and Pearson’s chi-squared tests were performed to test correlations. *n* = 20. Scale bar, 10 μm. *p value < 0.05

To further characterize the effect loss of *SIM2s* has on replicating cells, we analyzed MCF7-*shSIM2* and control cells during anaphase. Previously, it has been shown that loss of factors involved in HR contributes to sister-chromosome non-disjunction, or the inability for sister chromosomes to fully separate during mitosis. Traditional DNA staining is sufficient to reveal these abnormalities, which can present as DNA bridges, lagging strands, or acentric chromosomes (Fig. 3a). After synchronization using a di-thymidine block, cells were stained with Hoechst 33342 and analyzed for the presence of anaphase abnormalities. Here, we observed a significant increase in the fraction of cells containing DNA bridges and lagging strands but not acentric chromosomes in cells containing *shSIM2* (Fig. 3b–d).

### Loss of SIM2s decreases RAD51 recruitment

**Fig. 4:**
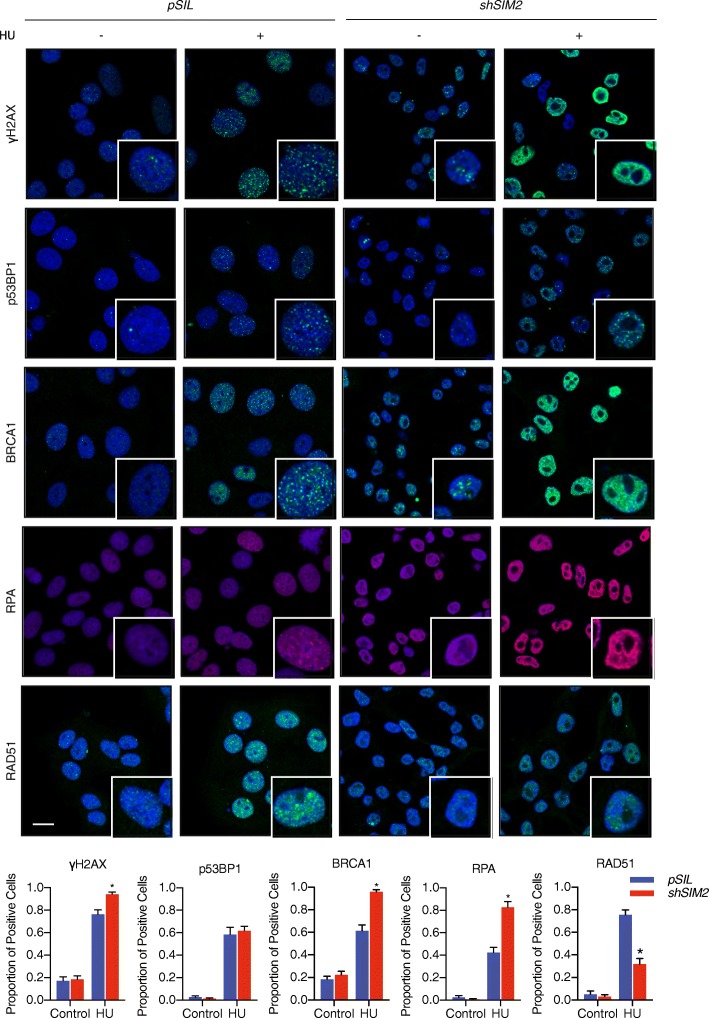
Loss of SIM2s leads to a decrease in RAD51 foci MCF7 cells. MCF7 cells containing *shSIM2* or *pSIL* were treated with 0.5 mM HU, fixed 24 h later, and finally probed with the indicated antibody. Cells containing 10 or more foci were considered to be positive for the factors indicated as has previously been demonstrated [13]. Scale bars, 10 μm. Values indicate the mean ± SE with *n* ≥ 100 cells. Student’s *t* test was performed to test significance. *p value < 0.05

Previous studies have demonstrated that there is significant overlap between DDR pathways and the stabilization and resolution of replication stress [24, 25]. With the increase in genomic instability associated with loss of SIM2, we sought to determine if loss of SIM2s correlates with a reduction in DDR factors. To start, we looked at γH2AX foci formation within the nucleus of MCF7-*shSIM2* and MCF7-*pSIL* cells. As a previous study has demonstrated that γH2AX levels rise between 16 and 48 h after HU treatment, we dosed our cells with 0.5 mM HU for 24 h before fixation and immunofluorescent staining [24]. Interestingly, we observed a significant increase in γH2AX foci in cells containing *shSIM2* that were treated with HU (Fig. 4). This finding is likely due to an increase in unresolved stalled replication forks and an increase in dsDNA breaks [13].

To further investigate how loss of *SIM2* leads to an increase in genomic instability, we next looked at the recruitment of BRCA1, a factor that has been shown to be crucial for the stabilization of stalled replication forks [10]. Interestingly, we observed an increase in BRCA1 recruitment in response to 24 h of 0.5 mM HU treatment (Fig. 4). Previous reports have demonstrated that increased levels of BRCA1 can be observed with loss of 53BP1 [26]. As such, we next assessed the recruitment of p53BP1 to sites of replication stress and found that loss of *SIM2* had no effect on p53BP1 recruitment (Fig. 4).

Having seen an increase in BRCA1 recruitment without observing a change in p53BP1, we hypothesized that loss of *SIM2* impedes processes downstream of BRCA1, and thus leads to an increase in BRCA1 levels as more BRCA1 peptides are recruited with few replication lesions being resolved. To continue to investigate where SIM2s resides in this pathway, we next looked at the recruitment of RPA to stalled replication forks, as it coats ssDNA and protects it for nucleolytic enzymes as well as prevents the formation of secondary DNA structures that would impede the repair process [27–29]. Interestingly, we observed an increase in the number of RPA-positive foci within MCF7 cells containing *shSIM2* after 24 h of treatment with 0.5 mM HU (Fig. 4).

Finally, having previously observed that loss of *SIM2* leads to a decrease in the formation of RAD51 subnuclear foci in response to ionizing radiation, we tested whether loss of *SIM2* also impeded RAD51 recruitment in response to replication stress. In contrast to other factors tested, loss of *SIM2* led to a significant decrease in RAD51 foci formation in MCF7 cells treated with 0.5 mM HU for 24 h (Fig. 4).

### SIM2s is necessary for RAD51 recruitment in response to genotoxic stress in primary mammary epithelial cells

**Fig. 5:**
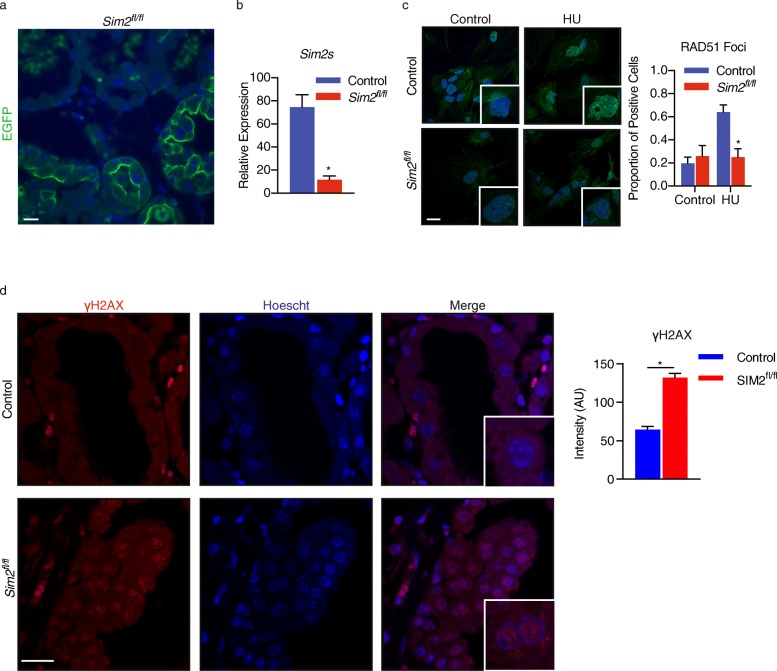
Loss of SIM2s in a mouse model decreases RAD51 recruitment and increases genomic instability. **a** Recombination of the SIM2 locus was confirmed in late-stage pregnant mice by the presence of eGFP. Scale bars, 20 μm. **b** RNA isolated from mammary glands of control or SIM2^fl/fl^ mice was analyzed via RT-qPCR for the presence of *Sim2s* mRNA. **c** MECs isolated from control and late-stage pregnancy SIM2^fl/fl^ mice were treated with 0.5 mM HU for 24 h before being assessed for the presence of RAD51 foci. Cells containing 10 or more foci were considered to be positive for the factors indicated as has previously been demonstrated [13]. Scale bars, 10 μm. **d** Mammary glands were collected from lactating SIM2^fl/fl^ and control mice and assessed for the presence of γH2aX. Quantification of γH2aX is nuclear γH2aX intensity minus background. Scale bars, 20 μm. Values indicate the mean ± SE with *n* = 3. Student’s *t* test was performed to test significance. *p value < 0.05

To confirm our finding that RAD51 is reduced in cells lacking SIM2s, we utilized a mammary tissue-specific conditional *Sim2* knockout mouse, which was generated via a “floxed” *Sim2*^*fl/fl*^ allele. *Sim2* is conditionally deleted for the duration of lactation by crossing *Sim2*^*fl/fl*^ mice with *Wap*^*Cre/+*^ mice, which express Cre recombinase under the control of the whey acidic protein (*Wap*) promoter. *Wap* is specifically expressed in mammary alveolar epithelial cells from mid-pregnancy through lactation, and thus allows for conditional knockout of *Sim2.* To visualize Cre recombinase activity, *Wap*^*Cre/+*^;*Sim2*^*fl/fl*^ (*Sim2*^*fl/fl*^) and Wap^Cre/+^;Sim2^+/+^ (control) mice were genetically tagged with Gt (ROSA)26Sor^tm4(ACTB-tdTomato,-EGFP)luo^/J (mTmG) [30]. Confirmation of efficient loss of the *Sim2s* locus after pregnancy was visualized in tissue sections using immunofluorescence and was confirmed via RT-qPCR (Fig. 5a, b). Primary mammary epithelial cells (MEC) were isolated from mice during late pregnancy (day 18) and treated with 0.5 mM HU or DMSO for 24 h prior to immunostaining for RAD51. As we observed in MCF7 cells, loss of *SIM2s* led to a significant reduction in RAD51 foci in cells treated with HU (Fig. 5c).

### Loss of SIM2s increases γH2AX levels in mammary tissue

With a significant decrease in RAD51 with the loss of *SIM2s*, we hypothesized that prolonged absence of SIM2s would lead to an increase in genomic instability, resulting in elevated levels of DNA damage. To test this, we isolated the fourth inguinal mammary glands of *Sim2*^*fl/fl*^ and control mice at lactation day 18, allowing MECs to progress through pregnancy and peak lactation; two stages that metabolically stress the mammary tissue and result in the elevation of factors associated with HR [31]. Sections were then probed for γH2AX. Loss of *SIM2* resulted in significantly higher intensities of γH2AX, suggesting that decreased levels of SIM2s result in elevated levels of genomic instability (Fig. 5d).

### SIM2s interacts with RAD51 and is necessary for RAD51 binding to the chromosome

**Fig. 6:**
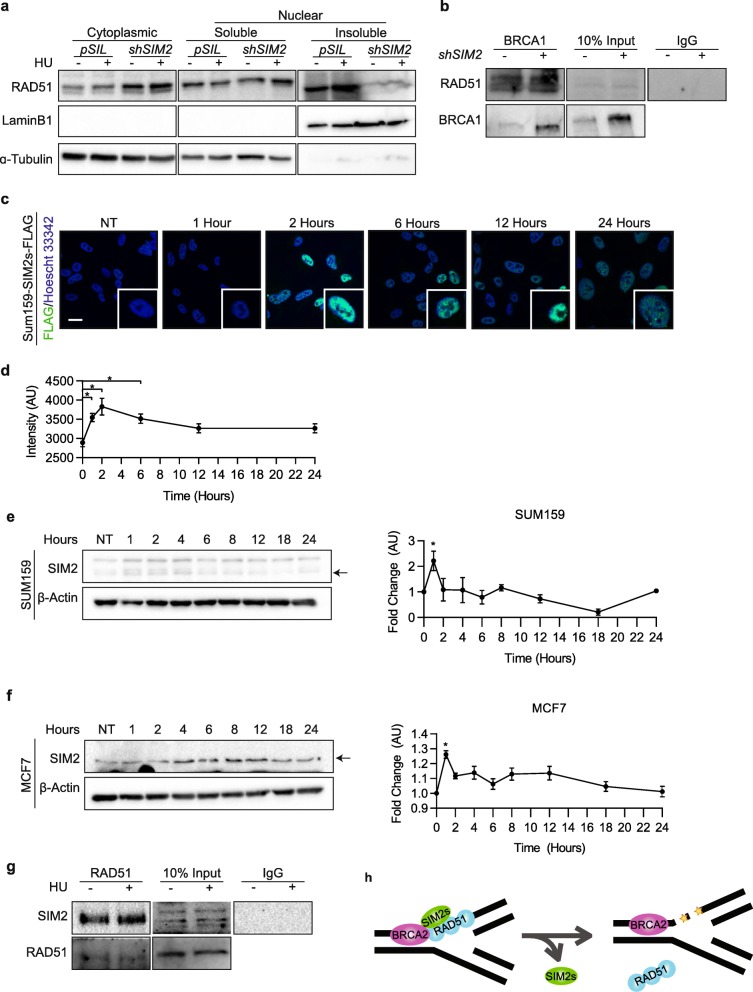
SIM2s interacts with RAD51 and is necessary for subnuclear RAD51 localization. **a** MCF7-*shSIM2* and MCF7-*pSIL* cells were treated with DMSO or 0.5 mM HU for 24 h and then fractionated before being probed for RAD51. α-Tubulin and LaminB1 were used as loading controls to verify efficient separation of fractions. **b** MCF7-*shSIM2* and MCF7-*pSIL* cells were treated with 0.5 mM HU and harvested 2 h later. BRCA1 was immunoprecipitated and lysates were probed for the indicated proteins. **c** Stabilization and localization of SIM2s was assessed in SUM159-*SIM2s-FLAG* treated with 0.5 mM HU and fixed at the indicated timepoints before being probed for FLAG. **d** Quantification of nuclear FLAG from **c**. **e**, **f** Western blot analysis of SIM2 stabilization in **e** SUM159-*SIM2s-FLAG* and **f** MCF7 cells in response to 0.5 mM HU treatment. Arrow indicates predicted molecular weight of SIM2s. Quantification is the ratio of SIM2s to β-actin. **g** RAD51 was immunoprecipitated in MCF7 cells after 2 h of treatment with HU or DMSO and lysates were probed for the indicated proteins. **h** Graphical representation showing fork stabilization in cells containing SIM2s and fork collapse with loss of SIM2s

It has been established that RAD51 translocates to the nucleus before binding to dsDNA breaks in response to DNA damage [32]. To test where in this process loss of SIM2s interferes with RAD51 loading, we isolated cytoplasmic, soluble nucleus, and insoluble nuclear (chromatin) fractions from MCF7-*shSIM2* and MCF7-*pSIL* cells that had been treated with DMSO or 0.5 mM HU for 24 h (Fig. 6a). Counterintuitive to the decrease in RAD51 foci we observed in response to DNA damage in the *shSIM2 cells*, we detected an increase in RAD51 levels in the cytoplasm, both basally and with treatment of HU. We also observed no change in the ability of RAD51 to translocate to the nucleus (Fig. 6a). However, loss of SIM2s led to a significant decrease in the levels of RAD51 found in the insoluble/chromatin fraction of the nucleus (Fig. 6a).

Having previously shown that SIM2s interact with BRCA1, we next hypothesized that SIM2s may be necessary for RAD51 to interact with other proteins within the repairosome [13]. However, MCF7 cells containing *shSIM2* that were treated with 0.5 mM HU did not show a significant decrease in the ability of RAD51 to bind to BRCA1, as observed through immunoprecipitation of BRCA1 (Fig. 6b). Interestingly, there appears to be an increase in BRCA1 protein levels in cells containing *shSIM2* (Fig. 6b). This finding is not typically observed with loss of RAD51 and may indicate a secondary pathway by which SIM2 regulates DNA-damage repair [33].

Based on this finding, we next hypothesized that SIM2s may directly interact with RAD51. As no previous studies show the kinetics of SIM2s in response to HU treatment, we first analyzed SIM2 levels in response to 0.5 mM HU treatment over time. In order to facilitate this process, *pLPCX-SIM2s-FLAG* was stably transduced into MCF7 cells, which resulted in cell senescence and death (data not shown). As such, *pLPCX-SIM2s-FLAG* was then transduced into SUM159 TNBC cancer cell line [13], which endogenously expresses low levels of *SIM2* (data not shown). Immunofluorescent analysis of FLAG in SUM159 cells overexpressing SIM2s-FLAG showed an increase in nuclear FLAG after treatment with 0.5 mM HU (Fig. 6c, d). These findings were also confirmed via western blot analysis in SUM159 and MCF7 cells, where we saw increased levels of SIM2s 2–4 h after treatment with 0.5 mM HU (Fig. 6e, f). Having shown that SIM2 levels peak 2 h after treatment with HU, we next immunoprecipitated RAD51 from MCF7 cells treated with DMSO or HU for 2 h and probed for SIM2s to test for their interaction. Interestingly, we observed that RAD51 interacts with SIM2s both basally and with HU treatment (Fig. 6g), confirming that SIM2 interacts with the RAD51 complex.

## Discussion

In this study, we have shown that loss of *SIM2s* sensitizes replication forks to genotoxic stress, leading to an increase in replication fork collapse (Figs. 1 and 2). This coincides with abnormal separation of sister chromatids during mitosis, which results in chromatin fragmentation and aneuploidy (Fig. 3) [7]. These findings parallel those previously observed with BRCA1 mutations, with familial BRCA1/2-associated tumors having a higher instance of DNA deletions and chromosomal translocations than sporadic tumors [34]. The rapid flux in genomic integrity that is observed in BRCA-mutated tumors predisposes them to mutations in *TP53*, estrogen receptor (ER), progesterone receptor (PR), and ERBB2 (HER2; human epidermal growth factor receptor 2), and thus biases them toward highly invasive, TNBC with a poor clinical prognosis [35].

A mutation in a single BRCA1/2 allele is sufficient to result in carcinogenesis; however, a single functional copy of BRCA1/2 is also sufficient to maintain HR functionality [34]. Thus, BRCA-mutated tumor progression is thought to predominantly occur through loss of heterozygosity (LOH). Yet, the mechanistic pathways underlying LOH are vague and revolve around accruing DNA damage. More recent studies have shown that replication stress is more sensitive to perturbations in BRCA1 levels than other established BRCA1 roles. More specifically, a mutation in a single copy of BRCA1 is sufficient to reduce replication fork stability [34]. This finding lends support to the notion that although the role BRCA plays in HR is crucial to maintaining genomic fidelity, the initial, and possibly more important increase in genomic instability seen during cancer progression in BRCA-associated tumors could be due to its role in maintaining replication fork stability. The rapid increase in genomic instability observed with *BRCA* mutations mimics those we see with loss of *SIM2s*, underpinning its importance in this pathway (Fig. 5).

The direct role SIM2s plays within this pathway remains unknown and requires further investigation. Here, we show that loss of *SIM2s* does not affect the ability of RAD51 to translocate to the nucleus in response to replication stress (Fig. 6). The ATP hydrolysis activity of RAD51 has drastically decreased during its evolution from RecA (the bacterial RAD51 homolog), allowing RAD51 paralogs to regulate RAD51 binding and unbinding to DNA [36]. RAD51 paralogs form two distinct complexes: RAD51B-RAD51C-RAD51D-XRCC2 (BCDX2 complex) and RAD51C-XRCC3, which require RAD51D and XRCC2 to catalyze RAD51 binding to DNA and the RAD51C-XRCC3 complex to catalyze RAD51 removal [37–40]. The loss of RAD51 foci in response to genotoxic stress with loss of *SIM2s* suggests that SIM2s may be interacting directly with RAD51 or indirectly acting on RAD51 through interaction/regulation of the BCDX2 complex. Interestingly, in colorectal cancer patients, where high levels of *SIM2s* are linked to poor prognosis, high levels of XRCC2 are also associated with poor clinical outcome [41]. This parallel may suggest that SIM2s is involved in the regulation of the BCDX2 complex.

In previous publications, we have demonstrated that loss of *SIM2s* in a xenograft model results in an EMT, characterized by decreased levels of E-Cadherin, increased activity of matrix- metalloproteinases (MMPs), and increased invasion and migration potential [13]. However, loss of *SIM2s* alone in a normal mammary gland is not sufficient to instigate tumor initiation (data not shown). It is not uncommon for tumor-suppressing factors to rely on a secondary mutation to initiate tumor development, and in fact, this trend is also observed in *BRCA1*, *BRCA2*, and *RAD51C* mutations [42–44]. Moreover, LOH in *TP53* in combination with mutation of any of these genes is sufficient to give rise to tumor cells [42–44]. As mentioned above, this combination results in drastic shift from cellular quiescence and toward TNBC [35].

Due to the strong association between BRCA mutations and early-onset breast carcinogenesis, genetic testing for BRCA1 and BRCA2 mutations has been suggested for individuals with breast cancer under the age of 60. However, an argument should be made to broaden the scope of genetic testing for individuals with early-onset breast cancer. In these individuals, multigene analysis of factors involved in DDR is warranted based on the correlation of elevated TNBC incidence and mutations in *BARD1*, *BRIP1*, *PALB2*, and the *RAD51* paralogs *RAD51C* and *RAD51D* [45]. The definitive role of RAD51 in alleviating replication stress, protecting damaged DNA from nucleases, and promoting genomic stability has long been established [46]. However, due to the embryonic lethality of *RAD51*^*−/−*^ and knockouts of *RAD51* paralogs, very little progress has been made toward understanding the regulation of RAD51 [47]. For example, RAD51C and XRCC3 have been known to play a role in HR for decades, but, due to the difficulty in researching genes that are critical for development, their involvement in replication fork restart has only recently been discovered [47]. This has also prevented the development of treatments directly targeting these mutations.

A unique therapeutic advantage of cancers with mutations in proteins involved in HR is their sensitivity to synthetic lethality treatments [48]. Two leading classes of drugs that have shown promising results are the platinum salts and PARPi. In fact, the PARPi Olaparib (AZD2281) has only recently gained approval by the Unites States Food and Drug Administration for use in BRCA-associated tumors [49]. These treatments aim to create dsDNA breaks either through crosslinking DNA, as in the platinum salts, or through the inhibition of PARP release from DNA, which forces DNA breaks during replication. These breaks could easily be repaired by cells with functional DDR but are lethal to cells with dysfunctional DDR pathways.

Although currently only approved for the treatment of BRCA-associated tumors, the efficacy of synthetic lethality treatments in cells with mutations in *SIM2s*, *XRCC2*, *RAD51*, and *RAD51C* has been shown by our lab and others [13, 41, 50]. Interestingly, RAD51 levels can be used as an indicator of the efficacy of PARPi treatments in breast cancer [51]. Moreover, BRCA-mutated tumors that express low levels of RAD51, and thus have low recombinase activity, have been shown to predict treatment efficacy [52–54]. This finding highlights the importance of fully understanding the factors involved in the regulation of HR and continuing to identify novel elements within this pathway, such as SIM2s. These efforts will ultimately lead to a better understanding of the intricacies involved in replication stress and improve patient outcomes.

## Conclusions

In summary, these findings support a role for SIM2s in the prevention of breast cancer progression through its integral part in maintaining genomic stability through DNA-damage repair and the resolution of replication stress. It has previously been established that dysregulation of RAD51 recruitment to sites of DDR is associated with a highly aggressive phenotype which can include lymph node recruitment, basal-like phenotypes, and TNBC status [55]. Contrary to previous reports, this association has been found to not only be due to the role RAD51 plays in DNA-damage repair, but also, more importantly, its pivotal role in the stabilization and resolution of stalled replication forks. Failure of a cell to sufficiently protect and resolve replication stress leads to a rampant increase in chromosomal abnormalities and cancer heterogeneity [56]. Thus, the characterization of the pathways associated with the maintenance of replication stability should be of great importance. In the data presented here, we have demonstrated that SIM2s is necessary for RAD51 to be loaded onto sites of replication stress, and in its absence, RAD51 is not recruited, leading to replication fork collapse.

## Supplementary information

**Additional file: 1 Figure S1.** Loss of *SIM2s* does not affect replication-fork restart time. (a-b) Visualization of CldU tract lengths from MCF7-*shSIM2* and MCF7-*pSIL* cells treated with DMSO or 10 mM HU. Table S1. Antibody List.

## Data Availability

The datasets used and/or analyzed during the current study are available from the corresponding author on reasonable request.

## Abbreviations

ATCC: American Type Culture Collection
ATM: Ataxia telangiectasia mutated
CldU: 5-Chloro-2′-deoxyuridine
DDR: DNA-damage repair
EMT: Epithelial mesenchymal transition
ER: Estrogen receptor
ERBB2: HER2; human epidermal growth factor receptor 2
HR: Homologous recombination
HU: Hydroxyurea
IdU: 5-Iodo-2′-deoxyuridine
IF: Immunofluorescence
LOH: Loss of heterozygosity
MEC: Mammary epithelial cell
MMP: Matrix metalloproteinases
PARPi: PARP inhibitor
PR: Progesterone receptor
RT-qPCR: Real-time qPCR
SIM2s: Singleminded-2s
TNBC: Triple-negative breast cancer
Wap: Whey acidic protein
