# Supplementary material for: Loss of SIM2s inhibits RAD51 binding and leads to unresolved replication stress
Source: Breast Cancer Res. 2019 Nov 27;21:125. doi: 10.1186/s13058-019-1207-z (PMC6882179; doi:10.1186/s13058-019-1207-z)
Supplement: Supplementary file 1 — Additional file: 1 Figure S1. Loss of SIM2s does not affect replication-fork restart time. (a-b) Visualization of CldU tract lengths from MCF7-shSIM2 and MCF7-pSIL cells treated with DMSO or 10 mM HU. Table S1. Antibody List. [file 13058_2019_1207_MOESM1_ESM.pdf]

Additional file 1:

Figure S1: Loss of *SIM2s* does not affect replication-fork restart time. (**a-b**) Visualization of CldU tract lengths from MCF7-*shSIM2* and MCF7-*pSIL* cells treated with DMSO or 10mM HU.

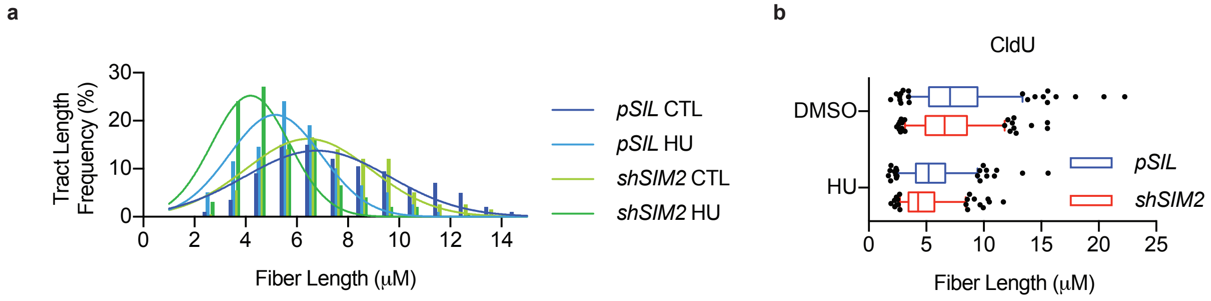

Table S1: Antibody List

Target	Manufacturer	Product Number	Dilution	Application
ATM	Abcam	AB32420	1:1000	WB
BRCA1	Abcam	AB131360	1:200<br>1:500	IF<br>WB
BRCA1	Abcam	AB16780	-	CO-IP
BrdU	BS-bioscience	347580	1:25	IF
BrdU	Abcam	Ab6326	1:400	IF
α-Tubulin	Thermo Fisher Scientific	A11126	1:500	WB
Lamin B1	Cell Signaling Technology	13435S	1:500	WB
p53BP1 (s1778)	Cell Signaling Technology	2675S	1:200	IF
γH2aX (s139)	Abcam	AB2893	1:200	IF
RPA	Abcam	Ab2175	1:200	IF
RAD51	Abcam	AB133534	1:200<br>1:500	IF<br>WB
RAD51	Abcam	AB1837	-	CO-IP
SIM2	Aviva	ARP38551_P050	1:500	WB
B-Actin	Cell Signaling Technology	3700S	1:5000	WB
γH2aX (s139)	Cell Signaling Technology	2577S	1:500	IF
Anti-Rabbit 2^{O}	Cell Signaling Technology	7074	1:2000	WB
Anti-Mouse 2^{O}	Cell Signaling Technology	7073	1:2000	WB
Alexa-488 goat anti-rabbit IgG	Life Technologies	A11034	1:1000	IF
Alexa-568 goat anti-mouse IgG	Life Technologies	A11004	1:1000	IF
